# Master Regulator SMC1A, Stabilized by N6‐Methyladenosine Reader IGF2BP1, Promotes HCC Progression Through Facilitating Enhancer–Promoter Interaction of Nestin

**DOI:** 10.1002/advs.75616

**Published:** 2026-05-22

**Authors:** Zhenxiang Peng, Diguang Wen, Lu Zeng, Lin Lv, Shengtao Liao, Wenguang Zhang, Zhechuan Mei, Chuanfei Li

**Affiliations:** ^1^ Gastroenterology The Second Affiliated Hospital of Chongqing Medical University Chongqing China; ^2^ Chongqing Municipal Health Commission Key Laboratory of Precision Diagnosis and Treatment of Liver Cirrhosis and Complications The Second Affiliated Hospital of Chongqing Medical University Chongqing China; ^3^ Gastroenterology Affiliated Banan Hospital of Chongqing Medical University Chongqing China

**Keywords:** enhancer, hepatocellular carcinoma, IGF2BP1, lipid nanoparticles, m6A, Nestin, SMC1A

## Abstract

HCC remains a leading cause of cancer‐related mortality, and effective therapies are limited. SMC1A, a core subunit of the cohesin complex involved in chromatin organization and transcriptional control, has not been fully characterized in HCC. SMC1A expression and prognostic value were analyzed using ICGC and single‐cell datasets, and validated in tissue microarrays and clinical specimens. Functional roles were examined in vitro, in vivo, and in patient‐derived organoids. Mechanistic studies combined transcriptomic, chromatin, and post‐transcriptional analyses to define downstream transcriptional regulation and upstream m6A‐dependent control. Therapeutic delivery was assessed using siRNA‐loaded LNPs. SMC1A was significantly upregulated in HCC and associated with poor prognosis. SMC1A knockdown suppressed proliferation, migration, invasion, and organoid growth, reduced tumor burden in xenograft and primary models, and promoted apoptosis. Nestin was identified as a transcriptional target of SMC1A; SMC1A facilitated enhancer–promoter interactions to activate Nestin transcription, and Nestin overexpression rescued malignant phenotypes after SMC1A depletion. Upstream, IGF2BP1 bound m6A‐modified regions within the SMC1A 3′‐UTR, stabilized SMC1A mRNA, and maintained the SMC1A–Nestin axis. Systemic LNP‐siSMC1A accumulated in the liver and inhibited tumor growth. SMC1A drives HCC progression through Nestin‐associated chromatin regulation and is maintained by IGF2BP1‐mediated m6A stabilization. LNP‐based silencing of SMC1A suppresses HCC.

## Graphical abstract

IGF2BP1‐mediated m6A stabilization sustains SMC1A expression, enabling cohesin‐associated chromatin regulation of Nestin in hepatocellular carcinoma. This work reveals an epitranscriptomic‐chromatin‐cytoskeletal regulatory axis linked to malignant phenotypes and identifies SMC1A as a biologically relevant vulnerability in HCC.

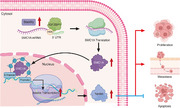

## 1 Introduction

Hepatocellular carcinoma (HCC) is the most common primary liver malignancy and remains a leading cause of cancer‐related mortality worldwide [1, 2]. Despite advances in surgical resection, locoregional interventions, and systemic therapies, long‐term survival remains poor, underscoring the need to identify novel molecular drivers and therapeutic targets in HCC [3, 4].

Structural maintenance of chromosomes protein 1A (SMC1A) is a core subunit of the cohesin complex, which regulates chromosome segregation, DNA repair, and higher‐order chromatin organization [5, 6]. By contributing to chromatin organization and promoter–distal regulatory communication, SMC1A participates in the transcriptional regulation of genes that control cell cycle progression and genome stability [7, 8, 9]. Consistent with these functions, dysregulation of the cohesin complex has been implicated in tumorigenesis [7, 10, 11]. Aberrant SMC1A expression has been reported in several malignancies, including breast, prostate, colorectal, and gastric cancers, and is associated with aggressive clinicopathological characteristics, drug resistance, and poor prognosis [7, 12, 13, 14, 15, 16, 17, 18, 19, 20]. However, the expression pattern, prognostic significance, and mechanistic roles of SMC1A in HCC remain incompletely characterized [21, 22].

Nestin, a class VI intermediate filament protein initially identified as a marker for neural stem/progenitor cells [23, 24], is re‐expressed in multiple cancers and is associated with increased cell motility, epithelial–mesenchymal transition, stem‐like properties, and enhanced angiogenic capacity [25, 26, 27, 28]. Elevated Nestin expression predicts poor clinical prognosis in multiple tumor types, including HCC [29, 30, 31, 32]. However, the upstream transcriptional regulators responsible for Nestin overexpression in liver cancer remain to be fully elucidated.

Post‐transcriptional regulation by N6‐methyladenosine (m6A) modification has emerged as a major determinant of mRNA metabolism, affecting transcript stability, translation efficiency, and degradation [33]. Among m6A reader proteins, Insulin‐like growth factor 2 mRNA‐binding protein 1 (IGF2BP1) binds m6A‐modified transcripts and stabilizes them, sustaining oncogenic gene expression programs [34, 35, 36, 37]. IGF2BP1 overexpression has been reported in several cancers, including HCC, where it is associated with aggressive tumor phenotypes [38, 39, 40]. Whether IGF2BP1–m6A–mediated regulation contributes to aberrant SMC1A expression in HCC remains to be elucidated.

Here, bulk and single‐cell transcriptomic analyses were integrated with clinical validation to investigate the expression pattern and prognostic relevance of SMC1A in HCC. Functional and mechanistic studies in vitro, in vivo, and in patient‐derived organoids were conducted to establish the oncogenic role of SMC1A, elucidate its transcriptional regulation of Nestin, and define its upstream control by m6A–IGF2BP1. Finally, lipid nanoparticle‐mediated delivery of small interfering RNAs (siRNAs) targeting SMC1A was evaluated as a therapeutic strategy for HCC.

## 2 Methods

### 2.1 Acquisition of Publicly Available Data

Gene expression profiles and corresponding clinical annotations for HCC were obtained from the International Cancer Genome Consortium (ICGC‐LIRI‐JP, LIRI‐JP project). Only cases with available survival and clinical information were included in the analysis. The dataset was accessed on June 13, 2025. Chromatin immunoprecipitation sequencing (ChIP‐seq) data (GSE76893) were retrieved from the Cistrome DB database. Other bioinformatic resources used for analysis included GEPIA2.0 (Gene Expression Profiling Interactive Analysis, version 2; http://gepia2.cancer‐pku.cn/), SRAMP (https://www.cuilab.cn/m6Asiteapp/old, used to predict putative m6A modification sites), RM2Target (http://rm2target.canceromics.org/, used to identify potential regulatory relationships between m6A‐related factors and target RNAs), RMVar (http://rmvar.renlab.org/, used to analyze potential binding regions). These platforms were primarily used for hypothesis generation and for prioritizing candidate regulators or modification sites for subsequent experimental validation.

### 2.2 Single‐Cell RNA Sequencing

Single‐cell RNA sequencing (scRNA‐seq) data were obtained from two publicly available datasets, GSE202642 and GSE290925. Raw count matrices from each dataset were processed independently using standard quality control procedures, including the removal of low‐quality cells and lowly expressed genes, followed by normalization and identification of highly variable genes. To reduce batch effects across datasets, an anchor‐based integration approach was used to combine datasets into a unified expression matrix for downstream analyses. Cell type annotation was performed based on the expression of canonical marker genes, supplemented by manual curation. For hepatocyte‐lineage cells, malignant HCC cells were distinguished from normal hepatocytes by integrating copy‐number variation (CNV) inference results. Cells exhibiting large‐scale chromosomal CNV were classified as tumor cells, whereas cells without significant CNV changes were annotated as normal hepatocytes.

### 2.3 Patients

Protein expression was assessed by Western blotting in tumor tissues and paired adjacent non‐tumorous liver tissues from five patients with HCC treated at the Second Affiliated Hospital of Chongqing Medical University. Tumor samples from two other patients were collected at the same institution for the generation of patient‐derived liver cancer organoids. All procedures were reviewed and approved by the Ethics Committee of the Second Affiliated Hospital of Chongqing Medical University. Tissue microarrays were obtained from AiFang Biotechnology (China) and analyzed by immunohistochemistry (IHC). The Scientific and Ethics Review Committee of AiFang Biotechnology approved the study protocol.

### 2.4 Western Blot

Total protein was extracted using lysis buffer, and protein concentration was determined using a BCA assay kit (Beyotime, China). Equal amounts of protein were denatured, separated by sodium dodecyl sulfate–polyacrylamide gel electrophoresis (SDS‐PAGE), and electrophoretically transferred onto polyvinylidene fluoride (PVDF) membranes (0.45 µm pore size, Millipore, Germany). Membranes were blocked with a buffer containing 5% skimmed milk (Beyotime, China) to reduce nonspecific binding and incubated overnight at 4°C with primary antibodies specific to target proteins. After washing, membranes were incubated with horseradish peroxidase (HRP)‐conjugated secondary antibodies for 1 h at room temperature. Protein bands were detected using enhanced chemiluminescence (ECL) and quantified using a chemiluminescence imaging system (Bio‐Rad, USA). Antibody information is provided in Table S1.

### 2.5 rAAV2/8

For hepatocyte‐specific knockdown of SMC1A in C57BL/6 mice, an adeno‐associated virus (AAV) vector carrying short hairpin RNA (shRNA) targeting SMC1A under the control of the liver‐specific TBG promoter was used. The recombinant virus, constructed by Heyuan Biotechnology (Shanghai, China), was diluted in phosphate‐buffered saline (PBS) to the appropriate concentration and administered via tail vein injection at 100 µL per mouse. Animals were maintained for 2–3 weeks after injection to allow shRNA expression and SMC1A knockdown before further experiments. The construct used was AAV‐shSMC1A#1: pAAV‐TBG‐MCS‐3xFLAG‐miR30shSMC1A#1‐WPRE.

### 2.6 Immunohistochemistry

Formalin‐fixed, paraffin‐embedded tissue sections were baked at 60°C for 3 h, deparaffinized in xylene, and rehydrated through a graded ethanol series. Antigen retrieval was performed in sodium citrate buffer (pH 6; Bioss, China) at 100°C for an appropriate duration using a standardized heat‐induced retrieval method. Endogenous peroxidase activity was quenched with hydrogen peroxide, followed by blocking of nonspecific binding with 5% goat serum. Sections were incubated overnight at 4°C with primary antibodies, followed by 1 h incubation at room temperature with HRP‐conjugated secondary antibodies. Visualization was performed with diaminobenzidine (DAB) substrate, and nuclei were counterstained with hematoxylin. After dehydration and mounting with neutral resin, stained sections were examined under a light microscope, and the intensity of IHC staining was scored. Immunohistochemical staining was independently assessed by two investigators in a blinded manner. A semi‐quantitative scoring system was applied based on both staining intensity and the proportion of positive cells. Staining intensity was scored as 0 (no staining), 1 (light yellow), 2 (brown‐yellow), and 3 (dark brown). The proportion of positive cells was scored as 1 (≤25%), 2 (26%–50%), 3 (51%–75%), and 4 (>75%). The final IHC score was calculated by multiplying the staining intensity score by the proportion score.

### 2.7 DEN/CCl4–Induced Primary HCC Model

Male C57BL/6 mice were administered a single intraperitoneal injection of diethylnitrosamine (DEN; 25 mg/kg in freshly prepared 0.9% NaCl) at postnatal day 14. After weaning, mice were maintained under standard conditions until 4 weeks of age, when chronic liver injury was induced by intraperitoneal injection of CCl_4_ in corn oil (1:3, v/v) twice weekly for 18 weeks (1 mL/kg injection volume, corresponding to 0.25 mL/kg CCl_4_). Control mice received an equal volume of vehicle injections. At 12 weeks after DEN administration, AAV was injected into the tail vein to induce liver‐specific gene silencing during the ongoing injury phase. All procedures were approved by the Ethics Committee of the Second Affiliated Hospital of Chongqing Medical University.

### 2.8 Sleeping Beauty Transposon‐Based AKT/N‐RAS‐Driven Primary HCC Model

Male C57BL/6 mice (6 weeks old) were injected with a plasmid mixture consisting of oncogene‐expressing constructs (AKT/N‐RAS) and a transposase‐encoding plasmid at a mass ratio of 4:1. Plasmids were dissolved in sterile PBS and administered at a total dose of 20–25 µg per mouse using a hydrodynamic injection volume of 0.1 mL/g body weight (approximately 10% of body weight). The mixture was administered by hydrodynamic tail vein injection over 5–7 s to promote efficient genomic transposition and hepatocyte expression. One week after plasmid administration, AAV was injected via the tail vein to induce liver‐specific gene modulation.

### 2.9 Hematoxylin and Eosin (H&E) Staining

Paraffin‐embedded liver tissue sections were baked at 60°C for 3 h, deparaffinized in xylene, and rehydrated through a graded ethanol series. Sections were stained with hematoxylin for 5–10 min and rinsed in running water until excess stain was removed. Sections were differentiated with 1% acid alcohol for a few seconds and blued in tap water. Cytoplasmic staining was performed using eosin for 1–3 min. Finally, the sections were dehydrated through a graded ethanol series, cleared in xylene, and mounted with neutral resin. Histological images were captured using a light microscope (Nikon, Japan).

### 2.10 Cell Lines and Culture Conditions

Human HCC cell lines Huh7 (RRID: CVCL_0336), MHCC97H (RRID: CVCL_4972), HepG2 (RRID: CVCL_0027), and Hep3B (RRID: CVCL_0326) were obtained from the Cell Bank of the Chinese Academy of Sciences (Shanghai, China). Huh7 and MHCC97H cells were cultured in Dulbecco's Modified Eagle Medium (DMEM; Gibco, USA) supplemented with 10% fetal bovine serum (FBS; PAN BIOTECH, USA). HepG2 and Hep3B cells were maintained in Minimum Essential Medium (MEM; Gibco, USA) supplemented with 10% FBS and non‐essential amino acids (NEAA; Gibco, USA). Cells were maintained at 37°C in a humidified incubator with 5% CO_2_, and culture medium was changed every other day. All cell lines used in this study were confirmed to be free of mycoplasma contamination.

### 2.11 Establishment of Stable Cell Lines

Cells were seeded in 6‐well plates and transduced at ∼50% confluence with lentiviral particles in the presence of 6–8 µg/mL polybrene. After 24 h, the culture medium was replaced with fresh complete medium. At 72 h post‐infection, puromycin (1–10 µg/mL) was added for 5–7 days to eliminate non‐transduced cells. Surviving stable populations were expanded and validated by Western blotting to confirm target knockdown or overexpression. Lentiviral constructs for SMC1A knockdown and overexpression were purchased from GeneChem (Shanghai, China), whereas the Nestin overexpression lentivirus was obtained from Heyuan Biotechnology (Shanghai, China).

shSMC1A#1: GCACUACAAGAAGCGUAAATT UUUACGCUUCUUGUAGUGCTT

shSMC1A#2: GCGGGAAAUUGAAGAGAAUTT AUUCUCUUCAAUUUCCCGCTT

OE‐SMC1A: Ubi‐MCS‐CBh‐SMC1A‐IRES‐puromycin

OE‐Nestin: PcSLenti‐EF1‐BSR‐CMV‐Nes‐3xFLAG‐WPRE

### 2.12 Cell Counting Kit‐8 (CCK‐8) Assay

Cells were seeded into 96‐well plates and allowed to adhere completely. Culture medium was replaced with 100 µL serum‐free DMEM containing 10 µL CCK‐8 reagent (MCE, USA) per well. Plates were incubated at 37°C with 5% CO_2_ for 2 h, and absorbance at 450 nm was measured using a microplate reader as an index of cell viability.

### 2.13 Cell Cycle Analysis

Cells were harvested by trypsinization, washed twice with cold PBS, and fixed overnight in 70% pre‐chilled ethanol. On the following day, samples were centrifuged to remove ethanol, washed with PBS, and incubated with propidium iodide (PI; 4A BIOTECH, China) staining buffer containing RNase in the dark for 30 min at room temperature. Cell cycle distribution was analyzed by flow cytometry, and data were processed using CytExpert software (Beckman Coulter).

### 2.14 Cell Apoptosis Assay

Both adherent and floating cells were collected, washed twice with cold PBS, and resuspended in pre‐chilled 1× Annexin V binding buffer. Annexin V–Alexa Fluor 647 and 4′,6‐diamidino‐2‐phenylindole (DAPI; 4A BIOTECH, China) were added, and cells were incubated in the dark for 15 min at room temperature. Apoptosis was quantified by flow cytometry, and early‐ and late‐apoptotic populations were determined using CytExpert software.

### 2.15 Transwell Migration and Invasion Assays

Cells were washed, resuspended in serum‐free medium, and seeded into the upper chambers of Transwell inserts (8 µm pore size; Jet Biofil, China). For migration assays, chambers were left uncoated, whereas for invasion assays, the upper surface of the membrane was coated with Matrigel (Corning, USA) according to the manufacturer's instructions. The lower chambers were filled with medium containing 10% FBS as a chemoattractant. After incubation at 37°C for 24–48 h, non‐migrated or non‐invaded cells were removed from the upper surface. Cells that migrated or invaded the lower surface were fixed with 4% paraformaldehyde, stained with 1% crystal violet (Solarbio, China), and counted under a light microscope in randomly selected fields.

### 2.16 Organoid Culture

Fresh HCC tissue samples were minced and enzymatically digested with collagenase IV (Sigma, USA) and DNase I under sterile conditions. The resulting cell suspension was filtered, centrifuged, and resuspended in organoid culture medium (AimingMed, China). Cells were mixed with Matrigel (Corning, USA) at a 1:1 ratio and seeded as domes in 24‐well plates. After polymerization at 37°C for 30 min, prewarmed culture medium was added. Media were refreshed every 2–3 days, and organoid structures typically formed within 5–10 days.

### 2.17 Lentiviral Transduction of Organoids

Organoids were first dissociated into small clusters using TrypLE Express (Gibco, USA). The organoids were then resuspended in organoid culture medium and transferred to ultra‐low attachment culture plates (Corning, USA). Lentiviral transduction was performed by centrifugation at 2000 rpm for 1 h at 32°C. Following spinfection, the organoids were incubated at 37°C in a humidified incubator with 5% CO_2_ for another 6–8 h. Subsequently, the organoids were collected and replated into multi‐well plates for further culture. After transduction, fresh organoid culture medium was replaced, and puromycin selection was initiated 48 h later to establish stable transduced organoids.

### 2.18 Subcutaneous Xenograft Tumor Model

Lentivirus‐stably transduced tumor cells were resuspended in serum‐free DMEM at a density of 1 × 10^6^–5 × 10^6^ cells per 100 µL. A total of 100 µL of the suspension was injected subcutaneously into the flank of 4‐week‐old male BALB/c nude mice. Tumor growth was monitored every few days, and mice were euthanized once tumors reached the predefined experimental endpoint. Tumor tissues were harvested for further analyses. All animal experiments were approved by the Ethics Committee of the Second Affiliated Hospital of Chongqing Medical University.

### 2.19 TUNEL Staining

Paraffin‐embedded tissue sections were deparaffinized, rehydrated, and subjected to antigen retrieval in citrate buffer using a high‐pressure heat‐induced retrieval method. After washing with PBS, sections were treated with Proteinase K and incubated with terminal deoxynucleotidyl transferase dUTP nick end labeling (TUNEL) reaction mixture (Solarbio, China) at 37°C for 1 h in the dark. Nuclei were counterstained with DAPI, sealed with neutral resin, and visualized under a fluorescence microscope (Nikon, Japan).

### 2.20 RNA Sequencing (RNA‐Seq)

Total RNA was extracted using TRIzol reagent, and RNA‐seq library preparation and sequencing were performed by Majorbio (China).

### 2.21 Quantitative Real‐Time PCR (qRT‐PCR)

Total RNA was isolated from cultured cells using TRIzol reagent and reverse‐transcribed into complementary DNA (cDNA) using a commercial reverse transcription kit according to the manufacturer's instructions. qRT‐PCR was carried out on the CFX96 Real‐Time System (Bio‐Rad, USA) using SYBR Green Master Mix (Takara, Japan). Gene expression was normalized to β‐actin, and relative transcript levels were calculated using the 2^−ΔΔ^ *^Ct^* method. Primer sequences are listed in Table S2.

### 2.22 Dual‐Luciferase Reporter Assay

Huh7 and MHCC97H cells with SMC1A or IGF2BP1 knockdown were seeded into 96‐well plates and cultured to approximately 70% confluence before transfection. Cells were co‐transfected with pRL‐TK and either wild‐type (WT) or mutant (MT) pGL3‐basic‐Nes‐promoter luciferase reporter plasmids (GeneCreate, China) using a transfection reagent. For assays targeting the SMC1A 3′‐UTR, cells were transfected with the pmirGLO‐SMC1A‐3′‐UTR construct. After 48 h, luciferase activity was measured using the Dual‐Luciferase Reporter Assay System (Promega, USA). Firefly luciferase signals were normalized to Renilla luciferase from pRL‐TK, and relative activity was compared between knockdown and control groups.

### 2.23 Electrophoretic Mobility Shift Assay (EMSA)

EMSA was performed with the LightShift Chemiluminescent EMSA Kit (Thermo Fisher Scientific, USA). Briefly, 5–10 µg of nuclear protein extract (NPE) was incubated with biotin‐labeled DNA probes and anti‐SMC1A antibody in binding buffer for 20 min at room temperature. Reaction mixtures were separated on 6% native polyacrylamide gels, transferred to membranes, UV crosslinked, and probed with streptavidin‐HRP. Signals were detected using chemiluminescence according to the manufacturer's protocol.

### 2.24 Chromosome Conformation Capture (3C) Assay

3C assays were performed following established protocols. Briefly, cells were crosslinked with 1% formaldehyde at room temperature, quenched with glycine, and then lysed for nuclear isolation. Crosslinked chromatin was digested with a restriction enzyme (EcoRI, Beyotime, China) and ligated under diluted conditions to favor intramolecular ligation. After reversal of crosslinks and DNA purification, interaction frequencies between selected genomic loci were quantified by quantitative PCR (qPCR), normalized to a control genomic region, and calculated using the 2^−Δ^ *^Ct^* method. Primer sequences and the corresponding enhancers with their IDs are listed in Table S3.

### 2.25 Assay for Transposase‐Accessible Chromatin (ATAC)

ATAC assays were performed to assess chromatin accessibility following standard protocols. Briefly, cells were harvested and lysed to isolate nuclei, which were subsequently incubated with Tn5 transposase to tag accessible chromatin regions. Transposed DNA was purified and analyzed by qPCR to assess accessibility at selected genomic loci. Primer sequences are listed in Table S4.

### 2.26 siRNA Transfection

Cells were seeded in 6‐well plates and transfected at ∼70% confluence with siRNAs (GenePharma, China) using HiPerFect Transfection Reagent (QIAGEN, Germany) following the manufacturer's instructions. After 48 h, cells were harvested for further experiments.

si‐IGF2BP1 sense: GGCUCAGUAUGGUACAGUAGA

si‐IGF2BP1 antisense UACUGUACCAUACUGAGCCAG

### 2.27 RNA Fluorescence In Situ Hybridization (FISH)

Cells grown on glass coverslips were fixed with 4% paraformaldehyde and permeabilized with Triton X‐100 (Beyotime, China). SMC1A mRNA was detected using an RNA FISH kit (GenePharma, China), while IGF2BP1 protein was visualized by immunofluorescence staining. Co‐localization was assessed using laser scanning confocal microscopy.

### 2.28 RNA Immunoprecipitation (RIP) Assay

Cells at ∼70% confluence were collected, and RIP was performed using the RIP kit (GeneCreate, China) following the manufacturer's protocol. RNA pulled down by the immunoprecipitation was reverse‐transcribed into cDNA, and target transcript enrichment was quantified by qRT‐PCR.

### 2.29 Agarose Gel Electrophoresis

Agarose was dissolved in 1× TAE buffer, cooled to ∼60°C, and supplemented with nucleic acid stain. Once solidified, the gels were placed in electrophoresis chambers containing 1× TAE buffer. DNA samples were mixed with 6× loading buffer at a ratio of 5:1 (v/v), loaded alongside a DNA ladder, and subjected to electrophoresis. Electrophoresis was continued until the tracking dye reached the desired position. Bands were visualized and documented under a UV transilluminator.

### 2.30 mRNA Stability Assay With Actinomycin D

Cells at approximately 70% confluence were switched to fresh complete medium and treated with actinomycin D (5 µg/mL; GLPBIO, USA) to inhibit transcription, and this time point was defined as 0 h. Cells were harvested at 0, 2, 4, 6, and 8 h, and total RNA was immediately extracted using lysis buffer supplemented with an RNase inhibitor (MCE, USA).

### 2.31 SELECT Assay

Site‐specific m6A modification on SMC1A transcripts was validated using the Epi‐SELECT m6A site identification kit (Epibiotek, China) with FTO‐assisted demethylation, according to the manufacturer's instructions. Briefly, total RNA was subjected to FTO treatment to remove m6A modification, followed by annealing of site‐specific upstream and downstream probes flanking the predicted m6A site. After probe hybridization, single‐base extension and ligation reactions were performed, and the resulting products were quantified by qPCR. Differences in threshold cycle (*Ct*) values between FTO‐treated and untreated samples were used to determine the presence of m6A modification at the candidate site. The probe and primer sequences used in this study are provided in Table S5.

### 2.32 Construction of Wild‐Type and m6A‐Site Mutant SMC1A 3′UTR Vectors

A WT pmirGLO vector containing the SMC1A 3′‐UTR was constructed, and a site‐specific mutant construct (MT3) was generated by mutating the third predicted m6A modification site within the SMC1A 3′‐UTR.

### 2.33 Immunofluorescence

Paraffin‐embedded sections were baked at 60°C for 3 h, deparaffinized in xylene, and rehydrated through a graded ethanol series. Multiplex immunofluorescence staining was performed using a TSA kit (AiFang, China) according to the manufacturer's instructions. Stained sections were examined and imaged using a fluorescence microscope.

### 2.34 Preparation of siRNA‐Loaded Lipid Nanoparticles (LNPs) via Microfluidic Mixing

LNPs encapsulating siRNA were generated using a rapid‐mixing microfluidic approach. 1,2‐Dimyristoyl‐rac‐glycero‐methoxy(polyethylene glycol)‐2000 (DMG‐PEG2000), cholesterol, and 1,2‐distearoyl‐sn‐glycero‐3‐phosphocholine (DSPC) were purchased from Macklin Biochemical (China), and DLin‐MC3‐DMA was obtained from Sunlipo Biotech (China). Lipids were dissolved in ethanol at a molar ratio of DLin‐MC3‐DMA/DSPC/cholesterol/DMG‐PEG2000 = 50/10/38.5/1.5, while 2′‐O‐methyl–modified (2′‐OMe) siRNA was dissolved in 25 mm acetate buffer (pH 4). The aqueous and ethanol phases were combined through a herringbone microfluidic chip (FluidicLab, China) at a volumetric flow rate ratio of 3:1 (v/v). The resulting mixture was dialyzed against PBS overnight to remove ethanol and adjust the pH to 7.4. LNP suspensions were sterilized with a 0.22 µm filter. Hydrodynamic diameter and polydispersity index were measured using a Zetasizer Nano ZS (Malvern Instruments, UK). Encapsulation efficiency was determined using a RiboGreen fluorescence assay by comparing total and unencapsulated siRNA following detergent‐mediated particle disruption. LNP–siRNA was administered via tail vein injection at a dose of 1 mg/kg siRNA.

### 2.35 In Vivo Fluorescence Imaging of LNPs

DiR (1,1′‐ Dioctadecyl‐3,3,3′,3′‐tetramethylindotricarbocyanine iodide) dye was incorporated into the lipid phase during LNP formulation. DiR‐labeled LNPs were injected into C57BL/6 mice via the tail vein. Fluorescence imaging was performed 12 h after injection under isoflurane anesthesia.

### 2.36 Orthotopic Xenograft Liver Tumor Model

Six‐week‐old male BALB/c nude mice were anesthetized with isoflurane. Luciferase‐expressing MHCC97H cells (2–3 × 10^5^ cells suspended in 20–30 µL DMEM: Matrigel, 1:1) were aseptically inoculated into the subcapsular region of the liver. At week 9, mice were injected via the tail vein with *D*‐luciferin potassium salt (150 mg/kg), followed by in vivo bioluminescence imaging.

### 2.37 Statistical Analysis

All statistical analyses were conducted using R software (v4.5.1) and GraphPad Prism (v8.0). For comparisons between two groups, an unpaired two‐tailed Student's *t*‐test was applied for data with an approximately normal distribution, whereas the Wilcoxon rank‐sum test was used for non‐normally distributed data. Paired data were analyzed using a paired Student's *t*‐test. Comparisons among three or more groups were performed using one‐way analysis of variance (ANOVA), followed by an appropriate multiple‐comparisons post hoc test. Dunnett's test was used when multiple experimental groups were compared with a single control group, whereas Tukey's multiple‐comparisons test was used for all pairwise comparisons. Categorical variables were analyzed using the chi‐square (*χ* ^2^) test or Fisher's exact test, as appropriate. Survival analysis was performed using the Kaplan–Meier method with the log‐rank test. Univariate and multivariable Cox proportional hazards regression models were applied to identify prognostic factors. Unless otherwise specified, all statistical tests were two‐sided, and a *p*‐value < 0.05 was considered statistically significant (*p < 0.05; **p < 0.01; ***p < 0.001; ns, not significant).

## 3 Results

### 3.1 SMC1A is Upregulated in HCC, Predicts Poor Prognosis, and Promotes Tumor Growth in Both Chemically and Genetically Induced Primary HCC Models

**FIGURE 1:**
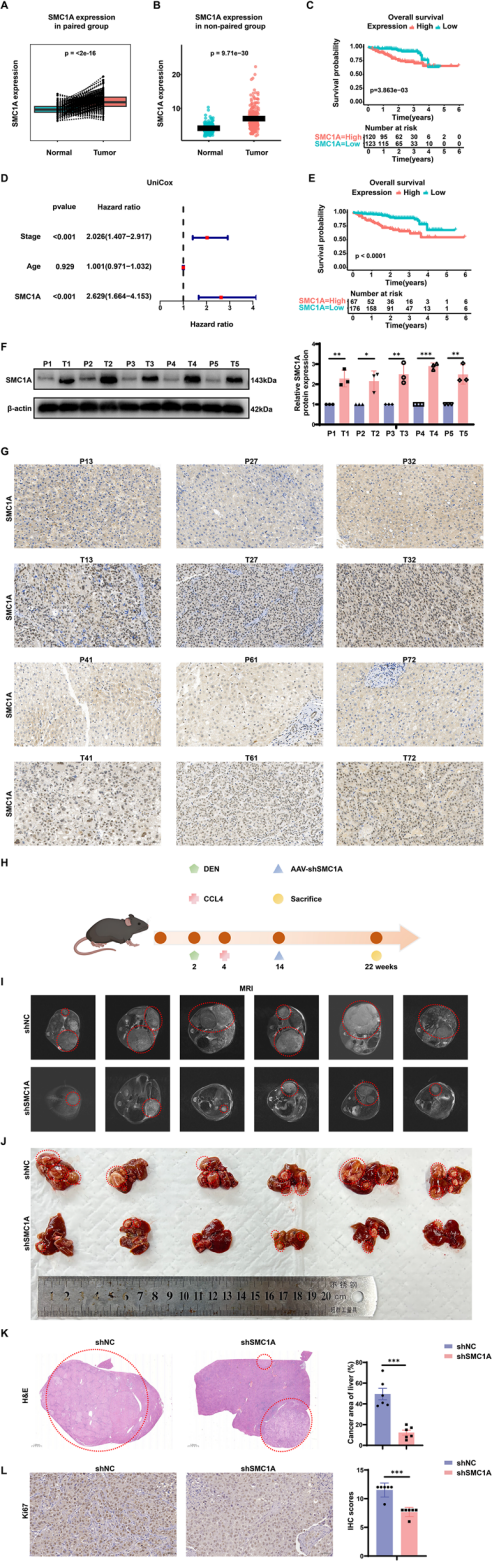
SMC1A is upregulated in HCC, and its silencing suppresses tumor growth in both chemically and non‐chemically induced primary liver cancer models. (A,B) Analysis of ICGC datasets showing SMC1A expression in paired and unpaired HCC and normal liver tissues. (C) Kaplan–Meier overall survival (OS) analysis in ICGC HCC patients, with high‐ and low‐expression groups defined by an optimal cutoff determined using the surv_cutpoint function in the “survminer” R package. (D) Univariate Cox regression analysis evaluating associations between SMC1A expression, age, tumor stage, and OS in ICGC HCC patients. (E) Kaplan–Meier OS analysis using the median SMC1A expression value as the cutoff. (F) Immunoblot analysis of SMC1A protein levels in five paired HCC and adjacent non‐tumor liver tissues. (G) Representative IHC images of SMC1A expression in a tissue microarray containing 80 paired HCC and adjacent non‐tumor samples. (H) Schematic of the DEN + CCl_4_‐induced primary HCC model and adeno‐associated virus (AAV)‐mediated SMC1A knockdown strategy. (I–K) Representative magnetic resonance imaging (MRI) scans, gross liver images with tumor area quantification, and H&E staining. (L) Representative IHC images and quantitative analysis of Ki‐67 expression in DEN + CCl_4_‐induced tumors.

Analysis of ICGC datasets showed that SMC1A expression was significantly higher in HCC tissues than in normal liver tissues, in both paired and unpaired samples (Figure 1A,B). Elevated SMC1A expression was significantly associated with poor patient prognosis (Figure 1C–E; Figure S1C). Single‐cell RNA‐sequencing data further revealed significantly higher SMC1A levels in HCC cells than in normal hepatocytes (Figure S1A,B). Immunoblotting confirmed consistently higher SMC1A protein levels in five paired HCC and adjacent non‐tumorous tissues (Figure 1F). Tissue microarray IHC of 80 paired HCC and adjacent non‐tumorous samples further demonstrated significantly higher SMC1A expression in tumor tissues (Figure 1G; Figure S1D,J).

To investigate the functional role of SMC1A in primary liver cancer development, a DEN + CCl_4_‐induced chronic liver injury model was established (Figure 1H). Twelve weeks after DEN injection, an AAV carrying shSMC1A#1 was delivered via the tail vein to specifically knock down hepatic SMC1A. Magnetic resonance imaging, gross liver morphology, and H&E staining showed that SMC1A silencing significantly suppressed tumor growth (Figure 1I–K) and reduced Ki‐67 expression in tumors (Figure 1L).

Non‐chemical etiologies, including chronic hepatitis C virus (HCV) and hepatitis B virus (HBV) infections, together with metabolic liver diseases, account for most HCC cases worldwide [41, 42]. Previous studies reported that coactivation of AKT and N‐RAS rapidly drives hepatocarcinogenesis by promoting abnormal hepatocyte proliferation and suppressing apoptosis [43, 44]. Accordingly, a non‐chemically induced HCC model was further established. The Sleeping Beauty transposon system was used to hydrodynamically deliver AKT and N‐RAS plasmids via tail vein injection (Figure S1E). One week later, AAV‐shSMC1A#1 was administered to silence hepatic SMC1A. As in the DEN + CCl_4_ model, silencing of SMC1A significantly suppressed tumor growth in the AKT/N‐RAS‐driven model (Figure S1F–H) and reduced Ki‐67 expression (Figure S1I).

These findings demonstrate that SMC1A is significantly upregulated in HCC and that its silencing inhibits tumor growth in both chemically and non‐chemically induced primary liver cancer models, supporting further investigation of SMC1A as a potential therapeutic target across diverse etiological contexts.

### 3.2 Knockdown of SMC1A Significantly Suppresses HCC Progression In Vitro and In Vivo

**FIGURE 2:**
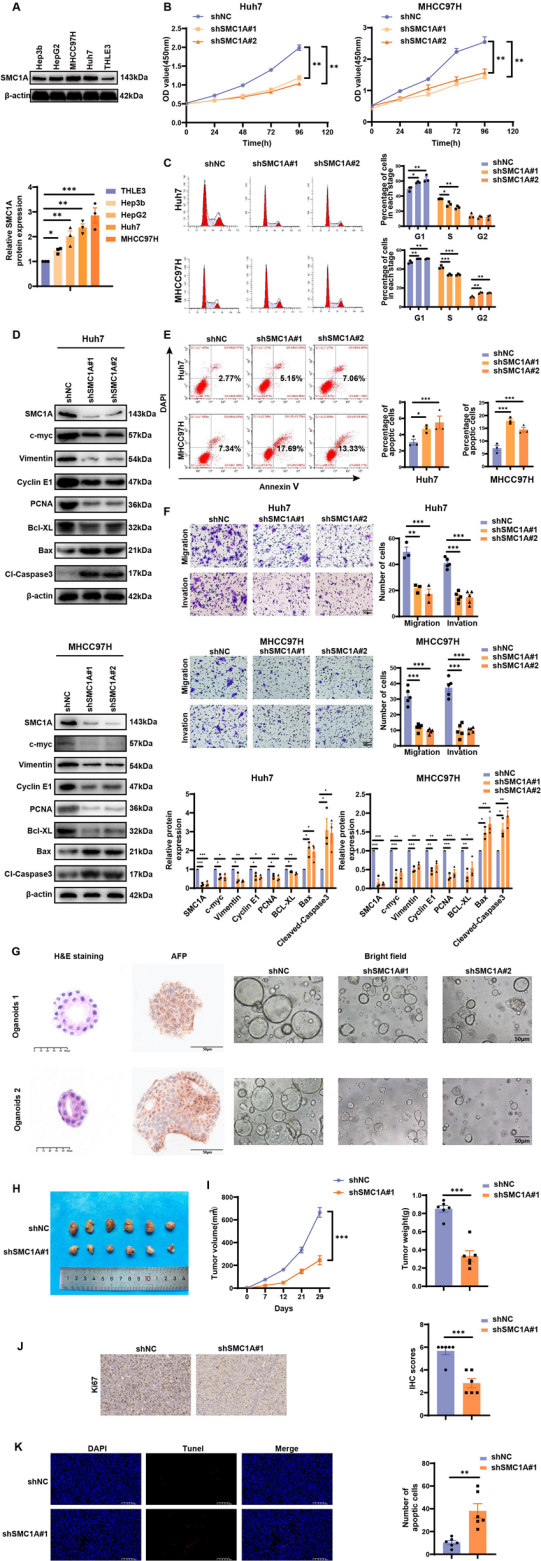
Knockdown of SMC1A suppresses proliferation, migration, and invasion in vitro, and inhibits tumor growth in vivo. (A) Western blot of SMC1A protein levels in THLE3 and four classical HCC cell lines. (B) Cell proliferation measured by CCK‐8 assays in Huh7 and MHCC97H cells. (C) Flow cytometric analysis of cell‐cycle distribution following SMC1A knockdown. (D) Immunoblot validation of SMC1A depletion and associated changes in proliferation‐, apoptosis‐, and EMT‐related proteins, including Bcl‐XL, PCNA, c‐Myc, Cyclin E1, Vimentin, Bax, and cleaved caspase‐3. (E) Quantification of apoptosis by Annexin V/DAPI staining and flow cytometry. (F) Representative images and quantification of Transwell migration and invasion assays following SMC1A silencing (n = 3 for migration in Huh7 cells; n = 5 for invasion and other Transwell assays). (G) Representative bright‐field images of organoid models validated by H&E and AFP staining. (H) Gross images of subcutaneous tumors from nude mice in control and SMC1A‐knockdown groups. (I) Final tumor weights and tumor growth curves were measured during the experimental period. (J,K) IHC staining for Ki‐67 and TUNEL in xenograft tumor sections. Data are presented as mean ± SEM. One‐way ANOVA with multiple‐comparison testing was applied.

The biological role of SMC1A in HCC was investigated through a series of in vitro and in vivo experiments. SMC1A expression was first assessed in the normal human liver cell line THLE3 and four classical HCC cell lines (Huh7, MHCC97H, Hep3B, HepG2). SMC1A expression was significantly higher in all four HCC cell lines compared with THLE3, with the highest levels in Huh7 and MHCC97H and the lowest in Hep3B (Figure 2A). Based on their relatively high endogenous SMC1A expression and robust proliferative capacity, Huh7 and MHCC97H cells were selected for subsequent loss‐of‐function analyses. Two independent lentiviral shRNA constructs were then generated to knock down SMC1A in Huh7 and MHCC97H cells, both effectively reducing SMC1A protein expression (Figure 2D). CCK‐8 assays demonstrated that SMC1A knockdown significantly inhibited cell proliferation (Figure 2B), while flow cytometric analysis revealed pronounced G1‐phase arrest (Figure 2C). Apoptosis was also significantly increased in SMC1A‐silenced cells (Figure 2E). Transwell migration and invasion assays further showed that SMC1A knockdown significantly impaired migration and invasion of HCC cells (Figure 2F). Western blotting corroborated these findings, showing reduced expression of the anti‐apoptotic protein Bcl‐XL, proliferation‐associated proteins c‐Myc and PCNA, the G1/S regulator Cyclin E1, and the metastasis marker Vimentin, together with increased expression of the pro‐apoptotic protein Bax and cleaved caspase‐3 (Figure 2D).

To further validate these observations, two patient‐derived HCC organoid models were established, with histological identity confirmed by H&E and alpha‐fetoprotein (AFP) staining. SMC1A knockdown significantly inhibited organoid growth (Figure 2G). In a subcutaneous xenograft model, silencing of SMC1A reduced tumor growth (Figure 2H,I), accompanied by lower Ki‐67 expression and increased apoptosis in the xenograft tissues (Figure 2J,K).

### 3.3 Overexpression of SMC1A Promotes HCC Progression In Vitro and In Vivo

**FIGURE 3:**
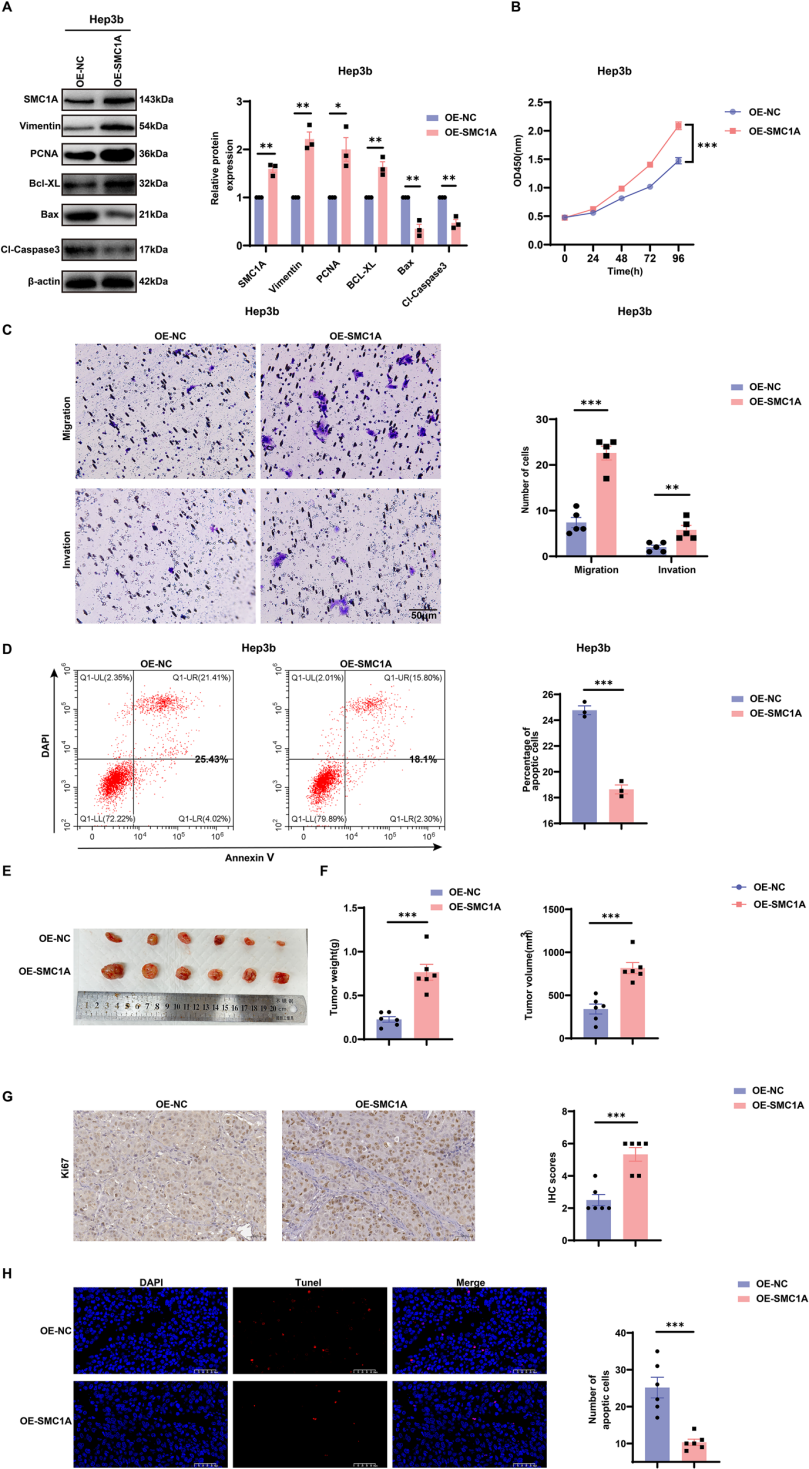
Overexpression of SMC1A promotes proliferation, migration, and invasion in vitro, and accelerates tumor growth in vivo. (A) Western blot confirming SMC1A overexpression in Hep3B cells and showing upregulation of Bcl‐XL, PCNA, and Vimentin, accompanied by downregulation of Bax and cleaved caspase‐3. (B) CCK‐8 assays assessing cell proliferation after SMC1A overexpression. (C) Representative images and quantification of Transwell migration and invasion assays (n = 5). (D) Flow cytometric analysis showing reduced apoptotic rates in SMC1A‐overexpressing cells. (E) Gross images of subcutaneous tumors from nude mice bearing control or SMC1A‐overexpressing cells. (F) Final tumor weights and tumor growth curves. (G,H) IHC staining for Ki‐67 and TUNEL in xenograft tumor sections.

To further determine its oncogenic potential, SMC1A was overexpressed in Hep3B cells (Figure 3A). CCK‐8 assays showed that SMC1A overexpression enhanced cell proliferation (Figure 3B), while Transwell assays revealed increased migration and invasion (Figure 3C). Apoptosis was significantly reduced following SMC1A upregulation (Figure 3D). Western blot analysis corroborated these results, showing increased levels of the anti‐apoptotic protein Bcl‐XL, the proliferation marker PCNA, and the metastasis‐associated marker Vimentin, together with reduced expression of the pro‐apoptotic proteins Bax and cleaved caspase‐3 (Figure 3A).

In vivo, subcutaneous xenograft experiments demonstrated that SMC1A overexpression significantly promoted tumor growth compared with control cells (Figure 3E,F). This was accompanied by increased Ki‐67 expression and reduced apoptosis in tumor tissues (Figure 3G,H). These findings indicate that SMC1A knockdown suppresses, whereas its overexpression promotes, proliferation, migration, invasion, and resistance to apoptosis in HCC cells in vitro, and enhances tumor growth with reduced apoptosis in vivo.

### 3.4 SMC1A Regulates HCC Progression Through Nestin

**FIGURE 4:**
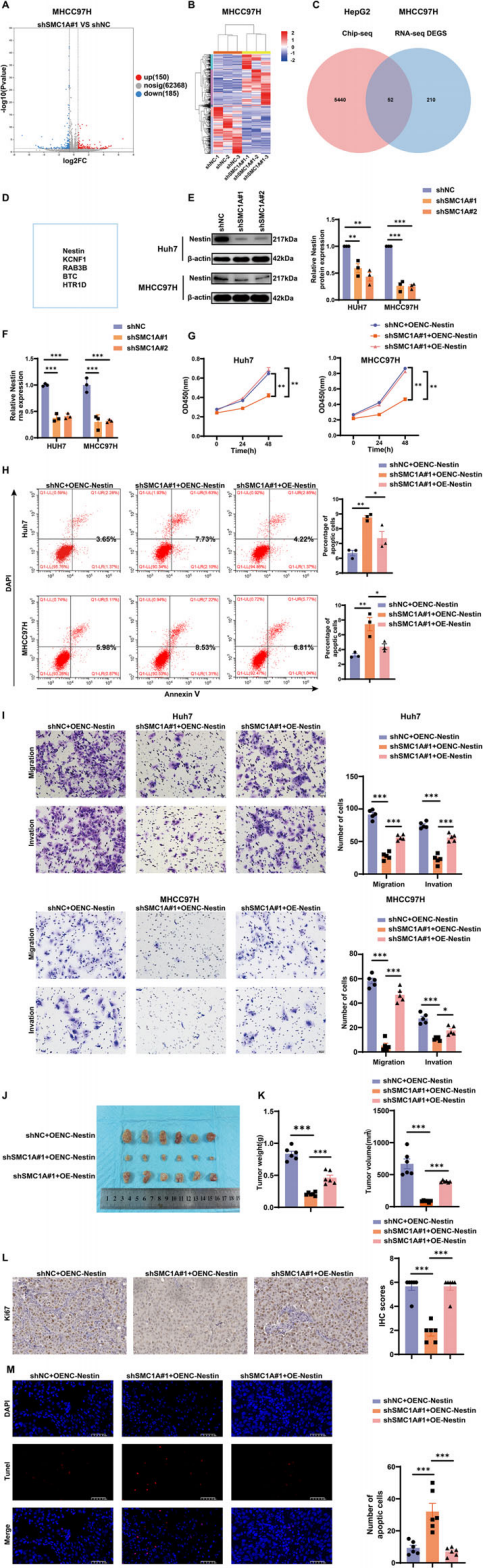
SMC1A regulates HCC malignant phenotypes through Nestin. (A,B) Volcano plot and heatmap showing DEGs between control and SMC1A‐silenced cells. (C) Venn diagram showing overlap between DEGs and the SMC1A ChIP‐seq dataset (GSE76893). (D) Identification of five overlapping candidate genes (NES, KCNF1, RAB3B, BTC, and HTR1D). (E,F) Immunoblotting and qRT‐PCR analyses of Nestin protein and mRNA expression. (G) CCK‐8 proliferation assays. (H) Flow cytometric analysis. (I) Representative images and quantification of Transwell migration and invasion assays (n = 5). (J,K) Gross tumor images, tumor volume curves, and final tumor weights of subcutaneous xenografts. (L,M) IHC staining for Ki‐67 and TUNEL in xenograft tumor sections.

To elucidate the mechanism underlying SMC1A‐mediated HCC progression, its role in transcriptional regulation was investigated. As a core component of the cohesin complex, SMC1A contributes to chromatin conformation remodeling and transcriptional regulation [7]. Based on these functions, it was hypothesized that SMC1A promotes malignant phenotypes by modulating transcription at the promoter regions of downstream target genes. Transcriptome sequencing of control and SMC1A‐silenced cells identified 335 differentially expressed genes (DEGs), including 150 upregulated and 185 downregulated (Figure 4A,B). Gene Ontology (GO) enrichment analyses showed that SMC1A silencing primarily affected transcriptional programs associated with cell proliferation, migration, angiogenesis, and vasculature development (Figure S2D). Intersecting these DEGs with a published SMC1A ChIP‐seq dataset from HepG2 cells yielded 52 overlapping genes (Figure 4C). Among these, only five (*Nestin, KCNF1, RAB3B, BTC*, and *HTR1D*) contained SMC1A‐binding sites within their promoter regions (Figure 4D).

Expression analysis of these five candidates using the GEPIA 2.0 database [45] demonstrated that Nestin alone was significantly upregulated in HCC and positively correlated with SMC1A expression (Figure S2A,B). Nestin, a class VI intermediate filament protein, has been reported to increase migration, invasion, stemness, angiogenesis, and unfavorable prognosis across multiple malignancies [25, 26, 28, 30], supporting its role as a potential downstream effector of SMC1A. Both qRT‐PCR and Western blotting confirmed that SMC1A silencing significantly reduced Nestin mRNA and protein levels (Figure 4E,F), indicating that SMC1A regulates HCC phenotypes at least partly through transcriptional activation of Nestin.

To confirm Nestin's functional relevance, rescue experiments were performed. Overexpression of Nestin restored the proliferative ability suppressed by SMC1A knockdown, as measured by CCK‐8 assays (Figure 4G), and reduced apoptosis induced by SMC1A depletion, as detected by flow cytometry (Figure 4H). Transwell assays revealed that Nestin overexpression reversed the migration and invasion deficits observed after SMC1A silencing (Figure 4I). In a subcutaneous xenograft model, Nestin upregulation partially rescued the inhibitory effect of SMC1A knockdown on in vivo tumor growth (Figure 4J,K), accompanied by increased Ki‐67 expression and decreased apoptosis in tumor tissues (Figure 4L,M). These findings demonstrate that SMC1A promotes proliferation, migration, invasion, and tumorigenesis in HCC by regulating Nestin.

### 3.5 SMC1A Promotes Nestin Transcription by Facilitating Enhancer–Promoter Chromatin Interactions

**FIGURE 5:**
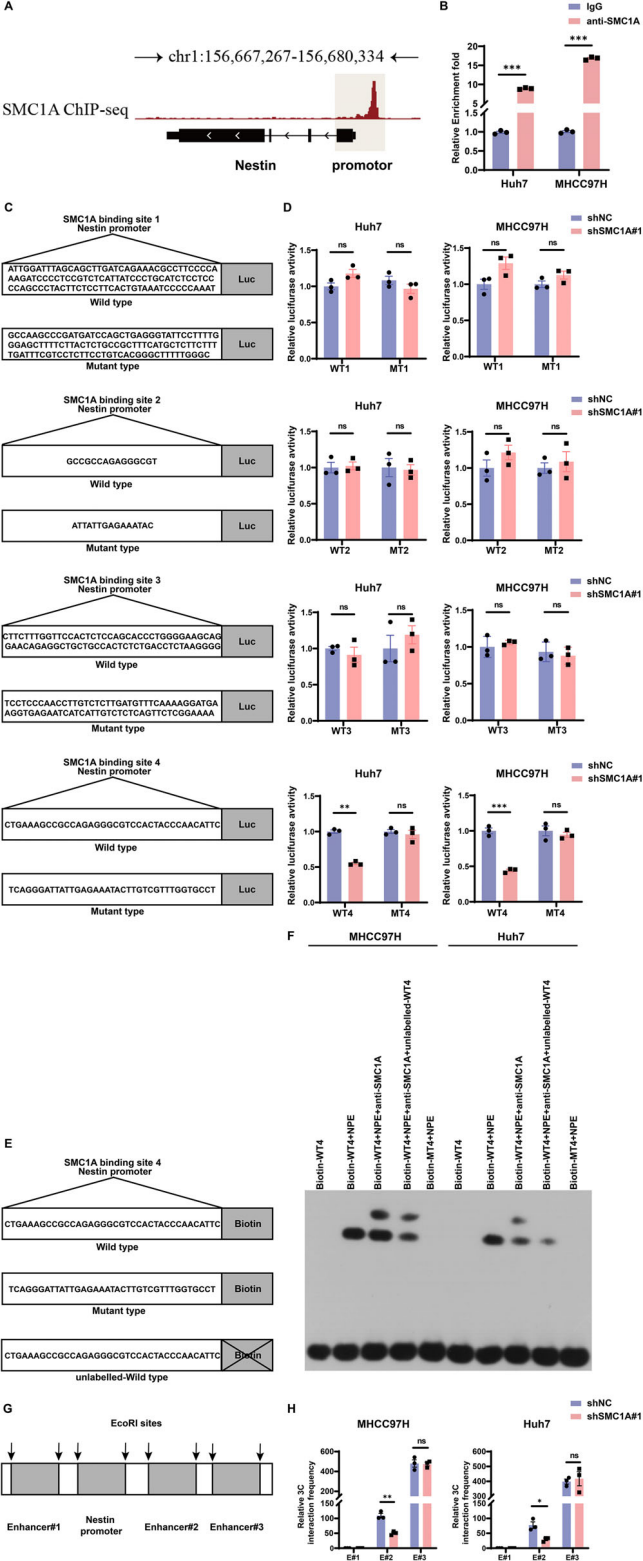
SMC1A promotes Nestin transcription by facilitating enhancer–promoter chromatin interactions. (A) SMC1A binding peaks at the Nestin promoter region. (B) CUT&RUN assays validating SMC1A occupancy. (C,D) Dual‐luciferase reporter assays. Relative luciferase activity was calculated as Firefly/Renilla and normalized to the corresponding shCtrl group for each construct. (E,F) EMSA performed in vitro. (G,H) 3C‐qPCR analysis showing interaction frequencies between the Nestin promoter and indicated enhancers in MHCC97H and Huh7 cells transduced with shNC or shSMC1A#1.

To further determine how SMC1A regulates Nestin expression and contributes to HCC progression, its interaction with the Nestin promoter was investigated. Analysis of the previously described ChIP‐seq dataset indicated that SMC1A bound to the Nestin promoter region in HepG2 cells (Figure 5A). This observation was validated in Huh7 and MHCC97H cells using cleavage under targets and release using nuclease (CUT&RUN) assays, which confirmed SMC1A enrichment at the Nestin promoter (Figure 5B). Predictions with the AnimalTFDB 3.0 database [46] identified four potential SMC1A‐binding sites within the promoter. A dual‐luciferase reporter assay demonstrated that the fourth site was required for SMC1A‐dependent transcriptional activation (Figure 5C,D). EMSA further provided in vitro evidence of the interaction between SMC1A and the fourth binding site (Figure 5E,F).

Given the established role of cohesin complexes in mediating chromatin architecture, the contribution of distal regulatory elements to SMC1A‐mediated Nestin transcription was examined. Candidate distal enhancer regions associated with Nestin were selected based on regulatory elements annotated in the GeneCards database. 3C‐qPCR analyses showed that several distal regions interact with the Nestin promoter; notably, only a subset of these interactions was significantly reduced following SMC1A knockdown, whereas others remained largely unchanged (Figure 5G,H; Figure S7A,B). These results indicate that SMC1A preferentially affects specific promoter‐associated chromatin interactions at the Nestin locus, rather than uniformly altering all detectable local contacts. ATAC‐qPCR showed no significant changes in chromatin accessibility at the Nestin promoter or distal enhancer regions upon SMC1A knockdown (Figure S2C), indicating that local chromatin openness is largely preserved. These results indicate that SMC1A activates Nestin transcription by facilitating enhancer–promoter chromatin interactions, consistent with a cohesin‐mediated regulatory mechanism.

### 3.6 SMC1A Expression is Maintained by IGF2BP1 Through m6A Modification in HCC

**FIGURE 6:**
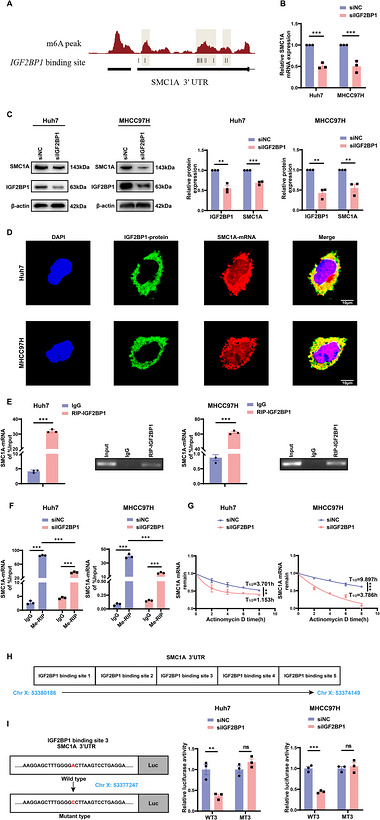
m6A‐dependent regulation of SMC1A expression by IGF2BP1 in HCC. (A) RMVar database prediction of potential IGF2BP1 binding sites within the SMC1A 3′‐UTR. (B,C) qRT‐PCR and immunoblot analyses of SMC1A expression following IGF2BP1 knockdown. (D) Combined fluorescence in situ hybridization and immunofluorescence analysis. (E) RNA immunoprecipitation (RIP) assay confirming IGF2BP1 binding to SMC1A mRNA. (F) Methylated RNA immunoprecipitation (MeRIP) showing reduced m6A enrichment of SMC1A mRNA after IGF2BP1 knockdown. (G) mRNA stability assays. (H) RMVar database prediction of five IGF2BP1 binding sites within the SMC1A 3′‐UTR. (I) Dual‐luciferase reporter assays validating IGF2BP1‐SMC1A interactions. Relative luciferase activity was calculated as Firefly/Renilla and normalized to the corresponding shCtrl group for each construct.

To explore the upstream regulatory mechanism driving SMC1A upregulation in HCC, SMC1A expression was confirmed to be consistently elevated in HCC tissues and cell lines (Figures 1F,G and 2A). Since m6A RNA modification has been widely implicated in tumorigenesis by regulating mRNA stability and oncogene expression [33, 34, 35, 36, 37], it was hypothesized that SMC1A may be regulated in an m6A‐dependent manner. Prediction using the SRAMP database identified multiple putative m6A modification sites within SMC1A mRNA (Figure S3A) [47, 48]. Further analysis of the RM2Target database identified 10 candidate m6A‐associated proteins that may regulate SMC1A [49]. To identify the key regulator, each of the ten proteins was silenced individually in Huh7 and MHCC97H cells. Immunoblotting showed that only IGF2BP1 knockdown resulted in a significant decrease in SMC1A protein levels (Figure 6C; Figure S3B). Analysis of the GEPIA2.0 database [45] revealed that IGF2BP1 expression was significantly elevated in HCC and positively correlated with SMC1A expression (Figure S4A,B), suggesting that IGF2BP1 plays a key role in regulating SMC1A. To further elucidate this relationship between IGF2BP1 and SMC1A, analysis of the RMVar database [50] indicated potential IGF2BP1‐binding regions within the 3′‐UTR of SMC1A mRNA (Figure 6A). IGF2BP1 silencing significantly reduced both SMC1A mRNA and protein levels (Figure 6B,C). As IGF2BP1 is an m6A “reader” protein that stabilizes methylated transcripts [34, 35, 36], it was hypothesized that IGF2BP1 maintains SMC1A expression by stabilizing its mRNA through m6A‐dependent mechanisms. FISH combined with immunofluorescence showed extensive co‐localization between IGF2BP1 protein and SMC1A mRNA in HCC cells (Figure 6D). RIP assays confirmed binding of IGF2BP1 to the SMC1A 3′‐UTR (Figure 6E). Methylated RNA immunoprecipitation (MeRIP) revealed reduced m6A enrichment signals for SMC1A transcripts following IGF2BP1 knockdown (Figure 6F). Furthermore, mRNA stability assays demonstrated accelerated degradation of SMC1A mRNA upon IGF2BP1 silencing (Figure 6G). RMVar‐based predictions identified five potential IGF2BP1 binding sites within the 3′‐UTR of SMC1A mRNA (Figure 6H). To validate these interactions, the 3′‐UTR was divided into 5 fragments, each cloned into a luciferase reporter plasmid. IGF2BP1 was shown to specifically bind to the third site, significantly increasing reporter gene expression. Mutation of this site abolished IGF2BP1 binding and enhancement of reporter activity, supporting the m6A‐dependent nature of this regulation (Figure 6I). To further validate m6A modification at this site, single‐nucleotide resolution SELECT assays were performed. FTO‐assisted SELECT analysis demonstrated a clear shift in qPCR‐C_T_ at this site following demethylation treatment, supporting the presence of m6A modification at this locus within SMC1A transcripts (Figure S4H). To confirm the requirement of this site for m6A‐dependent IGF2BP1 regulation, an SMC1A 3′‐UTR mutant construct targeting the third site was generated and compared with the corresponding wild‐type construct. MeRIP–qPCR analyses showed that mutation of this site significantly reduced m6A enrichment of the SMC1A 3′‐UTR in both Huh7 and MHCC97H cells (Figure S4D,E). IGF2BP1 RNA immunoprecipitation revealed significantly decreased binding of IGF2BP1 to the mutant SMC1A 3′‐UTR compared with the wild‐type construct (Figure S4F).

These results demonstrate that IGF2BP1 directly interacts with the m6A‐modified 3′‐UTR of SMC1A mRNA to stabilize the transcript. This mechanism maintains aberrant SMC1A overexpression in HCC, promoting its tumorigenic role.

### 3.7 m6A–IGF2BP1‐Mediated Regulation of SMC1A Maintains Nestin Expression in HCC

**FIGURE 7:**
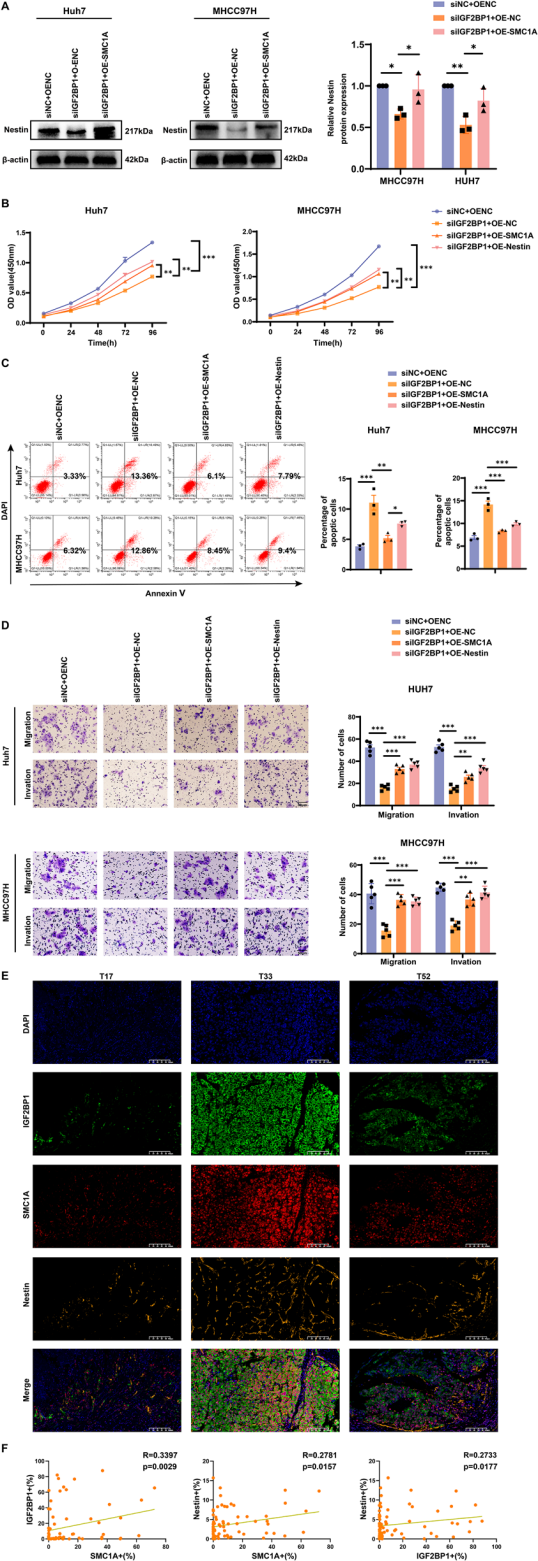
m6A‐IGF2BP1‐mediated regulation of SMC1A maintains Nestin expression and malignant phenotypes in HCC. (A) Immunoblot analysis in Huh7 and MHCC97H cells. (B) CCK‐8 proliferation assays. (C,D) Flow cytometry and Transwell migration/invasion assays. (E) Representative immunofluorescence images from an HCC tissue microarray (80 tumor and 80 adjacent non‐tumor samples). (F) Quantitative analysis showing significant positive correlations among SMC1A, IGF2BP1, and Nestin expression.

To elucidate the regulatory interplay among IGF2BP1, SMC1A, and Nestin in HCC, functional rescue experiments were conducted. Immunoblotting revealed that silencing IGF2BP1 significantly reduced Nestin expression, whereas SMC1A overexpression partially restored Nestin protein levels (Figure 7A). CCK‐8 assays showed that IGF2BP1 knockdown significantly inhibited HCC cell proliferation, and this effect was partially attenuated by overexpression of either SMC1A or Nestin (Figure 7B). Consistent results were observed in apoptosis and Transwell migration and invasion assays, in which ectopic expression of SMC1A or Nestin reduced apoptosis and partially restored the migratory and invasive abilities of HCC cells inhibited by IGF2BP1 knockdown (Figure 7C,D). Furthermore, immunofluorescence staining of a tissue microarray containing 80 HCC and 80 adjacent non‐tumorous samples demonstrated significant pairwise positive correlations among SMC1A, IGF2BP1, and Nestin expression (Figure 7E,F). These findings indicate that IGF2BP1 increases SMC1A expression by stabilizing its mRNA, and SMC1A, in turn, promotes Nestin expression, maintaining the malignant phenotype of HCC cells.

### 3.8 Therapeutic Silencing of SMC1A via LNP Delivery Attenuates HCC Progression

**FIGURE 8:**
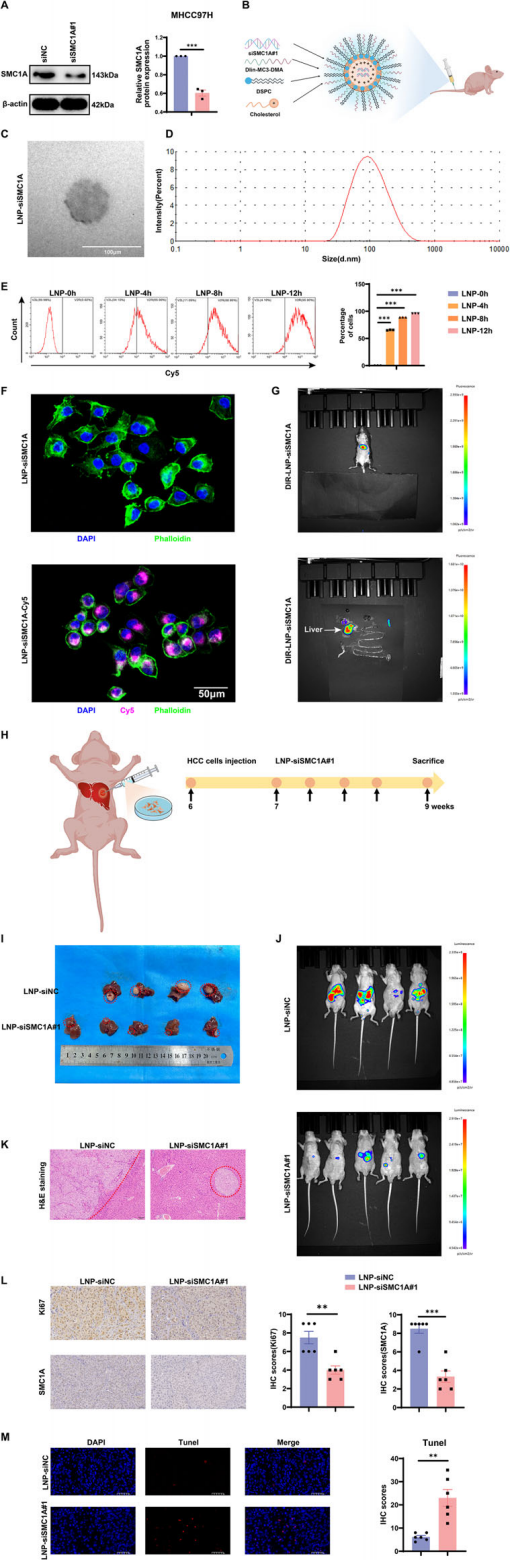
LNP‐mediated delivery of siSMC1A suppresses HCC progression in vivo. (A) Knockdown efficiency of siSMC1A#1 in HCC cells determined by immunoblotting. (B) Schematic diagram of siRNA‐loaded LNP preparation. (C,D) Transmission electron microscopy images of LNP morphology and measurements of hydrodynamic diameter and polydispersity index using dynamic light scattering. (E,F) Flow cytometry and immunofluorescence demonstrating intracellular siRNA delivery by LNPs. (G) In vivo near‐infrared fluorescence imaging showing hepatic accumulation of DiR‐labeled LNPs following systemic administration. (H) Schematic diagram of the orthotopic HCC xenograft model. (I) Gross liver images from each treatment group. (J) Bioluminescence imaging of tumor burden in orthotopic models treated with control or LNP‐siSMC1A#1. (K) H&E staining of tumor tissues. (L,M) IHC staining for Ki‐67 and TUNEL.

LNPs have recently emerged as an effective nucleic acid drug delivery platform due to their favorable biocompatibility, ability to encapsulate siRNAs, and high in vivo delivery efficiency [51]. In liver diseases, LNPs use the unique vascular architecture of the liver, including fenestrated sinusoidal endothelium, to achieve passive hepatic targeting and have shown promise in preclinical gene therapy studies [52, 53]. After confirming the knockdown efficiency of siSMC1A#1 (identical to that used in the lentiviral construct) in HCC cells (Figure 8A), siRNA‐loaded LNPs were synthesized (Figure 8B). Transmission electron microscopy and dynamic light scattering (DLS) confirmed the successful preparation of nanoparticles with a mean diameter of ∼100 nm (Figure 8C,D), consistent with the optimal size range for hepatic delivery [54]. Other physicochemical properties of the LNPs are provided in Table S6, with values consistent with those of well‐established lipid nanoparticle systems. Flow cytometry and immunofluorescence analysis demonstrated efficient intracellular delivery of siRNA (Figure 8E,F).

Histological examination of major organs, including the heart, liver, spleen, lung, and kidney, revealed no overt pathological abnormalities in mice treated with LNP‐siSMC1A#1 compared with PBS or LNP‐siNC controls (Figure S6A). Serum biochemical analyses showed that liver function parameters (ALT, AST) and renal function indicators (urea and creatinine) remained within the normal range, with no significant differences among the groups. These results indicate that systemic administration of LNP‐siSMC1A#1 did not induce hepatic or renal toxicity, suggesting no in vivo toxicity within the scope of this study.

Near‐infrared fluorescence imaging using DiR‐labeled LNPs confirmed hepatic accumulation after systemic tail vein injection (Figure 8G). Accordingly, the dosing regimen and administration frequency used in the subsequent in vivo experiments were based on previously published studies [55, 56, 57]. Based on these findings, an orthotopic xenograft model of HCC was established in nude mice using luciferase‐expressing HCC cells. Intravenous administration of LNP‐siSMC1A#1 significantly inhibited tumor growth (Figure 8H). Bioluminescence imaging revealed a significant (>10‐fold) reduction in signal intensity compared with controls (Figure 8J). Gross liver examination and H&E staining confirmed significant tumor regression (Figure 8I,K). IHC further showed reduced Ki‐67 expression and increased TUNEL‐positive nuclei, indicating suppressed tumor cell proliferation and enhanced apoptosis in orthotopic tumors treated with LNP‐siSMC1A#1 (Figure 8L,M).

## 4 Discussion

Over the past decade, the epidemiological and therapeutic landscape of HCC has undergone significant changes. Viral etiologies such as HBV and HCV now coexist with metabolic‐associated causes, including nonalcoholic steatohepatitis (NASH) and metabolic dysfunction‐associated fatty liver disease (MAFLD). Molecular subtyping has become increasingly refined, and combination therapies integrating immune checkpoint inhibitors with multi‐target tyrosine kinase inhibitors have improved survival in a subset of patients. However, clinical benefit remains heterogeneous, suggesting that the plasticity of epigenetic and post‐transcriptional regulation must be integrated into disease mechanisms and therapeutic strategies [58]. Multi‐omics analyses have further demonstrated that aggressive and stem‐like HCC states often coincide with chromatin remodeling and RNA modification networks [58, 59]. Within this framework, this study describes a hierarchical oncogenic axis in which the m6A reader IGF2BP1 stabilizes SMC1A transcripts, while SMC1A activates Nestin transcription by promoting enhancer–promoter interactions, linking nuclear transcriptional control with cytoskeletal remodeling and malignant phenotypes. This positions epitranscriptomic regulation, chromatin/cohesin dynamics, and cytoskeletal remodeling as a mechanistic continuum with translational relevance in difficult‐to‐treat HCC.

As a core component of the cohesin complex, SMC1A regulates three‐dimensional chromatin architecture and enhancer–promoter communication, amplifying transcriptional programs that control proliferation, metabolism, and survival [60, 61, 62]. In cancer, this can work as transcriptional rewiring, whereas partial cohesin dysfunction in other contexts contributes to genomic instability and tumor evolution [5]. Previous studies in HCC have shown that elevated SMC1A expression and phosphorylation are associated with increased proliferation and migration [22]. Consistent with the transcriptomic profiling data, enrichment analyses showed that SMC1A silencing primarily affected gene programs associated with proliferation, migration, and angiogenesis. These findings provide a broader transcriptomic framework for the subsequent identification and validation of key downstream targets of SMC1A.

Here, integration of RNA‐seq data with public ChIP‐seq datasets identified Nestin as a candidate transcriptional target of SMC1A. Nestin overexpression rescued proliferation, migration, invasion, and survival defects induced by SMC1A silencing. CUT&RUN and EMSA confirmed SMC1A binding at the Nestin promoter, while dual‐luciferase reporter assays demonstrated promoter activation, consistent with previous studies showing SMC1A‐mediated transcriptional activation. Indeed, previous studies have reported that SMC1A associates with the promoter regions of multiple genes and enhances their transcriptional activity. For example, SMC1A has been shown to bind the SNAIL promoter and promote its transcriptional activation [63]; similarly, SMC1A‐mediated promoter binding and transcriptional activation have been reported for SULT2B1 [14]. Although these studies did not systematically investigate whether SMC1A cooperates with distal regulatory elements, they established a consistent promoter‐level transcriptional activating role for SMC1A. Given the established role of SMC1A as a core component of the cohesin complex involved in higher‐order chromatin organization, the contribution of candidate distal regulatory elements to SMC1A‐mediated Nestin transcription was examined.

Beyond promoter occupancy, expanded 3C–qPCR analyses showed that although multiple distal candidate regions exhibited measurable contacts with the Nestin promoter, only a subset of these interactions was significantly reduced upon SMC1A depletion. This indicates that SMC1A promotes the maintenance of specific enhancer–promoter contacts at the Nestin locus rather than exerting a uniform effect on all detectable local interactions. ATAC‐qPCR analysis showed no significant changes in chromatin accessibility at either the Nestin promoter or the distal enhancer regions following SMC1A knockdown. This observation is consistent with previous studies reporting that perturbation of cohesin components, including SMC1A, can alter transcriptional output and regulatory interactions without inducing overt changes in local chromatin accessibility [9, 64, 65, 66, 67]. Together, these findings support a model in which cohesin‐mediated transcriptional regulation is primarily achieved through modulation of chromatin architecture rather than changes in accessibility alone.

As a core component of the cohesin complex, the regulatory specificity of SMC1A may not necessarily depend on recognition of a specific DNA sequence [68], but may instead involve promoter occupancy and broader chromatin regulatory interactions. In the present study, the expanded analysis of multiple candidate distal regulatory regions across the Nestin locus provided a broader locus‐level assessment of promoter‐associated chromatin contacts and strengthened support for a selective regulatory effect of SMC1A at this locus. These findings extend the functional landscape of SMC1A beyond chromosome maintenance, highlighting its role as a transcriptional hub linking three‐dimensional genome organization to phenotypic plasticity in HCC. One limitation to acknowledge is that the distal regulatory regions examined in this study were selected from candidate elements associated with the Nestin locus. More comprehensive analyses of chromatin architecture may therefore further refine the regulatory landscape of this locus in future work.

In line with this, accumulating evidence indicates that cohesin occupancy and function at promoters and enhancers are shaped by transcription factors and co‐regulatory complexes. For example, transcription factors such as SP1 and NFYA, as well as chromatin regulators including WDR5, physically associate with cohesin and stabilize its binding at shared promoter and enhancer sites [69]. Moreover, Mediator–cohesin coupling has been proposed to connect enhancer‐bound transcriptional regulators with the core promoter machinery, thereby supporting enhancer–promoter DNA looping [70]. These findings provide a mechanistic precedent that SMC1A/cohesin‐dependent transcriptional activation may involve coordinated actions with promoter‐associated transcription factors or co‐regulatory complexes.

Nestin, a class VI intermediate filament typically expressed during development and tissue regeneration, is reactivated in oncogenic reprogramming and in response to cellular stress. Beyond its role as a marker, Nestin integrates with vimentin and actin networks to enhance adhesion turnover, protrusion dynamics, and motility. Nestin can modulate focal adhesion signaling and invasion by regulating the spatial localization and activation of phosphorylated FAK (pFAK), coupling intermediate filament remodeling to adhesion turnover and invasive behavior [71]. Consistent with this cytoskeletal scaffold role, Nestin depletion has been associated with altered integrin and adhesion signaling, with functional consequences for matrix remodeling and invasion [71]. Nestin expression is associated with epithelial–mesenchymal transition (EMT), stemness, DNA damage tolerance, metabolic rewiring, angiogenesis, and immune evasion [24, 25, 26, 27, 28, 29, 30]. In particular, Nestin has been reported to be required for TGF‐β1–driven EMT responses, providing a basis for its association with enhanced motility and invasiveness in diverse cancer contexts [72]. Moreover, Nestin loss has been associated with cell‐cycle perturbation and increased apoptosis, supporting a broader role for Nestin in maintaining malignant cells under stress [73]. Elevated Nestin is associated with poor differentiation, vascular invasion, recurrence, and poor survival [29, 74]. In HCC, Nestin upregulation has been associated with EMT‐associated phenotypes and chemoresistance, and Nestin depletion can reverse these features in experimental settings [75]. In this study, Nestin was co‐upregulated with SMC1A in patient samples, and rescue assays identified Nestin as a key downstream effector translating SMC1A‐driven transcriptional activity into measurable oncogenic phenotypes. The Nestin‐associated phenotypes observed in this study are consistent with the previously reported functional roles of Nestin described above. These features suggest that SMC1A–Nestin signaling may have potential utility for IHC‐ and immunofluorescence‐based patient stratification.

m6A is a reversible and dynamic RNA modification implicated in proliferation, apoptosis, microenvironmental remodeling, and stemness, making it a therapeutically tractable pathway [33, 76, 77]. While methyltransferase “writers” and demethylase “erasers” establish the m6A landscape, reader proteins confer functional outcomes. YTH family proteins generally promote decay or translation, whereas IGF2BP proteins stabilize oncogenic transcripts [35, 78, 79]. Most previously reported m6A‐dependent oncogenic networks in HCC act by modulating the stability or translation of individual transcripts [80, 81, 82], exerting comparatively limited effects on downstream signaling. The IGF2BP1–SMC1A axis identified here extends m6A regulation from a transcript‐centric mode to chromatin architecture–dependent transcriptional amplification, enabling coordinated activation of broader gene programs. Nestin represents a functionally relevant output of this regulatory layer, serving as a cytoskeletal and stemness‐associated factor that integrates transcriptional amplification to enhance cellular plasticity, proliferation, and invasive behavior in HCC. Crosstalk between m6A and other epigenetic or post‐translational modifications (such as histone methylation, ubiquitination, or SUMOylation) can synergistically enhance tumor‐promoting programs [83]. Consistent with enrichment of m6A within the 3′‐UTR near stop codons, functional mapping revealed a regulatory region in the SMC1A 3′‐UTR bound by IGF2BP1. Knockdown of IGF2BP1 reduced SMC1A transcript half‐life and protein abundance, placing epitranscriptomic regulation within the HCC driver network. By stabilizing SMC1A, a core component of the cohesin complex, this regulatory axis links m6A‐dependent RNA regulation to transcriptional amplification mediated by chromatin architecture, representing a mechanistically distinct mode of m6A action in HCC. These findings highlight disruption of the IGF2BP1–SMC1A interaction as a potential strategy to reduce Nestin‐mediated tumor progression.

Accumulating evidence indicates that IGF2BP1 upregulation is driven by multiple oncogenic signals. IGF2BP1 has been identified as a transcriptional target of Wnt/β‐catenin signaling and may act as a downstream effector within Wnt‐regulated gene programs, [84]. MAPK/ERK signaling cooperates functionally with IGF2BP1 to promote tumor progression, whereas mTORC2 signaling has been shown to recruit IGF2BP1 into the MYC‐associated survival program [85, 86, 87]. Furthermore, hypoxic conditions induce IGF2BP1 expression in a HIF‐1α‐dependent manner, and IGF2BP1 is tightly associated with MYC family‐driven oncogenic networks, including MYCN‐dependent feedforward regulatory loops [88, 89]. These signaling pathways establish a coherent upstream regulatory framework that accounts for IGF2BP1 overexpression in HCC.

Given that immune checkpoint inhibitor (ICI)–based combinations (e.g., atezolizumab–bevacizumab) and multi‐kinase/VEGFR tyrosine kinase inhibitors (TKIs; e.g., lenvatinib and sorafenib) now represent central systemic therapies for advanced HCC, it is reasonable to consider whether the IGF2BP1–m6A–SMC1A–Nestin axis may also shape therapeutic response and resistance [90, 91]. SMC1A‐driven chromatin remodeling, together with a Nestin‐associated stem‐like and cytoskeletal program, could promote phenotypic plasticity and adaptive persistence, facilitating escape from kinase inhibition or immune pressure. In parallel, cohesin‐associated regulation of genome stability and DNA damage signaling intersects with innate immune sensing pathways, including cGAS–STING and interferon signaling, which are known to modulate ICI responsiveness. In this context, elevated SMC1A expression, by buffering replication stress and DNA damage responses, may contribute to a less inflamed tumor state in some contexts [92]. Immune‐excluded HCC subsets, classically associated with Wnt/CTNNB1 activation, exhibit intrinsic resistance to ICIs, underscoring that tumor‐intrinsic transcriptional programs can gate antitumor immune responsiveness [93, 94]. Moreover, accumulating evidence indicates that epitranscriptomic regulators directly participate in immune regulation in liver cancer models. m6A readers and RNA‐binding proteins such as YTHDF1 and IGF2BP1 have been associated with immunosuppressive phenotypes and reduced benefit from PD‐1 blockade, providing a mechanistic basis through which an IGF2BP1‐centered regulatory circuit could couple RNA metabolism to immune escape [56, 95, 96]. Finally, because m6A regulatory machinery has been implicated in TKI resistance and epithelial–mesenchymal transition programs in HCC, future studies should determine whether SMC1A overexpression correlates with poorer responses to lenvatinib or sorafenib and whether combined targeting of the IGF2BP1–SMC1A axis can sensitize tumors to TKIs and/or ICIs [97].

Further studies are warranted to refine the broader downstream signaling architecture associated with SMC1A‐dependent transcriptional regulation in HCC. In conclusion, this study identifies a mechanistically integrated oncogenic axis in HCC progression, in which IGF2BP1 stabilizes SMC1A transcripts in an m6A‐dependent manner, and SMC1A promotes Nestin transcription by facilitating enhancer–promoter chromatin interactions, driving aggressive tumor behavior. These findings establish SMC1A as a central node linking epitranscriptomic regulation, chromatin architecture, and cytoskeletal remodeling, and support further investigation of SMC1A as a potential therapeutic target in HCC.

## Author Contributions

Chuanfei Li, Zhechuan Mei, Zhenxiang Peng, Diguang Wen, and Wenguang Zhang designed the study. Zhenxiang Peng, Diguang Wen, and Lu Zeng conducted the experiments. Zhenxiang Peng drafted the manuscript and prepared the figures; Lin Lv and Shengtao Liao performed the statistical analyses. All authors reviewed and approved the final manuscript. Zhenxiang Peng and Diguang Wen contributed equally to this work.

## Funding

This work was funded by the National Natural Science Foundation of China (Grant Number: 82103206), General Project of Chongqing Natural Science Foundation (Grant Number: CSTB2025NSCQ‐GPX0367), Kuanren Talents Program of the Second Affiliated Hospital of Chongqing Medical University (Grant Number: kryc‐yq‐2224) and Open Project (General Project) of the Key Laboratory of Chongqing Municipal Health Commission for Science and Technology Joint Medical Research (Grant Number: 2026KFXM047), the Medical Science and Technology Research Program of Chongqing Banan Science and Technology Bureau and Chongqing Banan Health Commission (Grant No.BNWJ202300101), Natural Science Foundation of Chongqing (CSTB2023NSCQ‐MSX0784).

## Ethics Statement

All procedures were reviewed and approved by the Ethics Committee of the Second Affiliated Hospital of Chongqing Medical University.

## Consent

Informed consent was obtained from all individual participants included in the study.

## Conflicts of Interest

The authors declare no conflicts of interest.

## Supporting information




**Supporting File 1**: advs75616‐sup‐0001‐FiguresS1‐S7.zip.

**Supporting File 2**: advs75616‐sup‐0002‐TableS1‐S6.docx.

## Data Availability

All publicly available datasets analyzed in this study have been properly cited in the Methods and Results sections.
