# Supplementary figures and images for: Master Regulator SMC1A, Stabilized by N6‐Methyladenosine Reader IGF2BP1, Promotes HCC Progression Through Facilitating Enhancer–Promoter Interaction of Nestin
Source: Adv Sci (Weinh). 2026 May 22;13(43):e75616. doi: 10.1002/advs.75616 (PMC13336101; doi:10.1002/advs.75616)

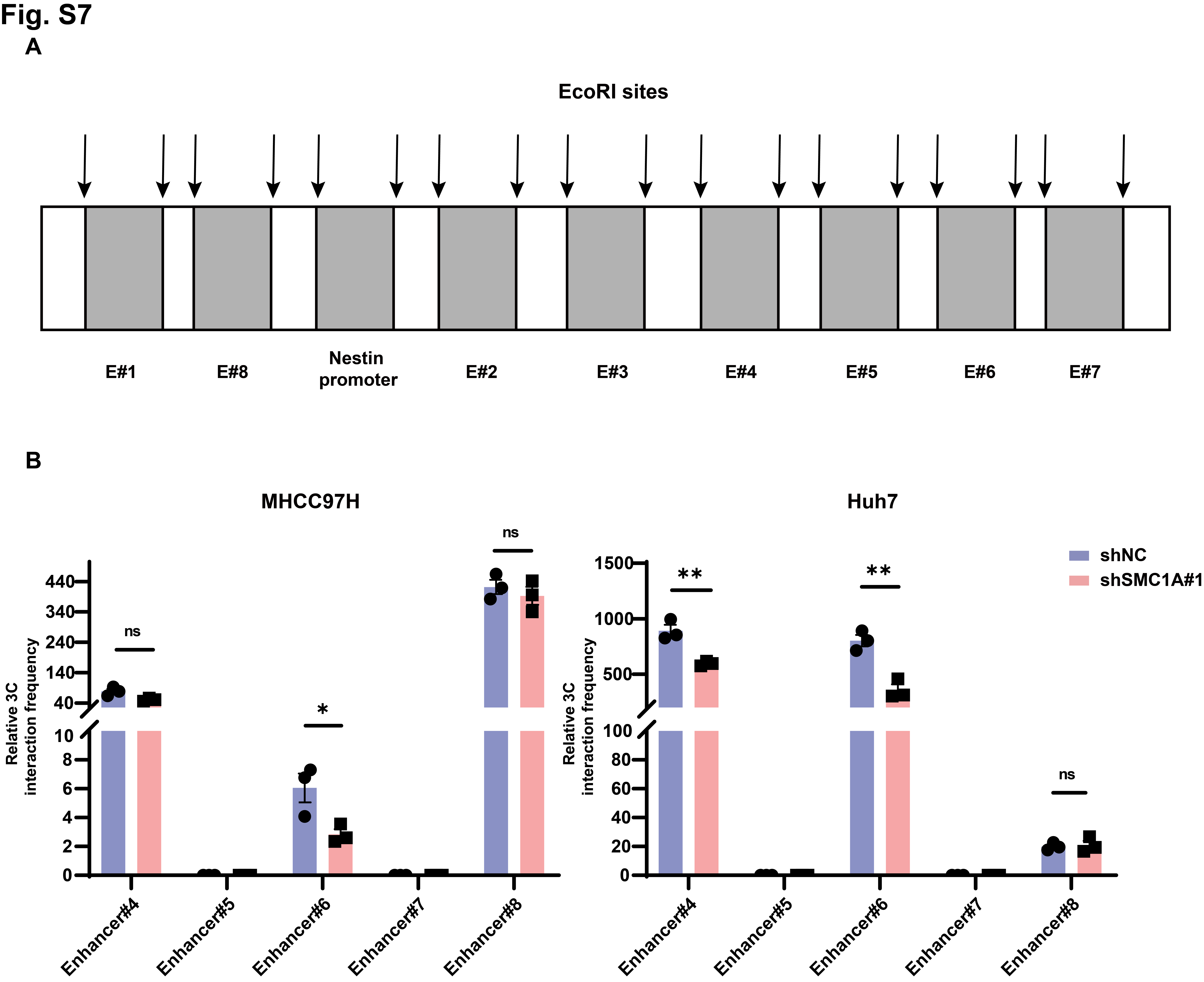

Supplement: Supplementary file 1 — Supporting File 1: advs75616‐sup‐0001‐FiguresS1‐S7.zip. [file ADVS-13-e75616-s002.zip › Supplementary Fig.7.jpg]

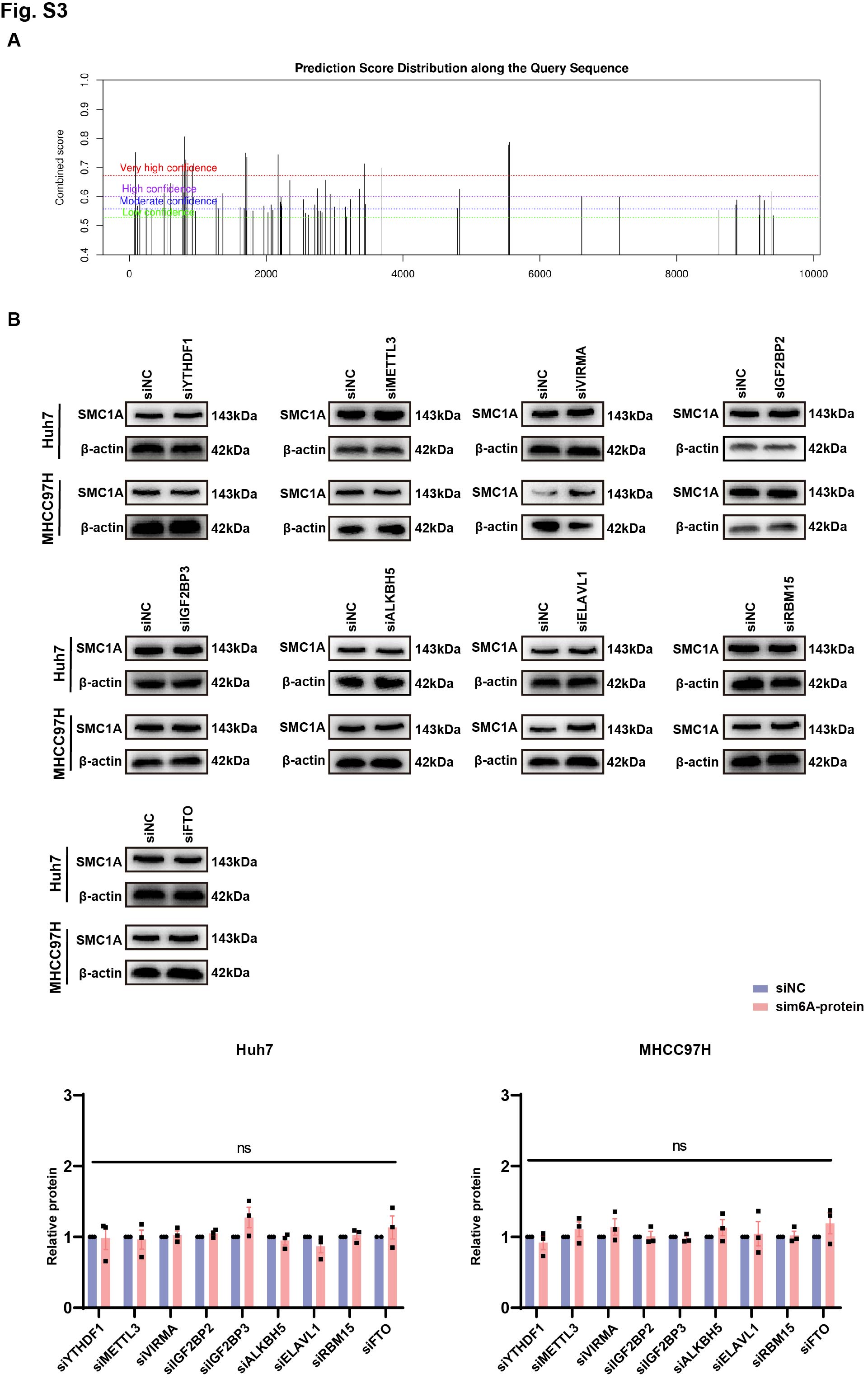

Supplement: Supplementary file 1 — Supporting File 1: advs75616‐sup‐0001‐FiguresS1‐S7.zip. [file ADVS-13-e75616-s002.zip › Supplementary Fig.3.jpg]
