# Supplementary material for: Master Regulator SMC1A, Stabilized by N6‐Methyladenosine Reader IGF2BP1, Promotes HCC Progression Through Facilitating Enhancer–Promoter Interaction of Nestin
Source: Adv Sci (Weinh). 2026 May 22;13(43):e75616. doi: 10.1002/advs.75616 (PMC13336101; doi:10.1002/advs.75616)
Supplement: Supplementary file 2 — Supporting File 2: advs75616‐sup‐0002‐TableS1‐S6.docx. [file ADVS-13-e75616-s001.docx]

**Table S1. Antibody Information**

| **Antibody** | **Producer** | **Application** |
| --- | --- | --- |
| SMC1A | ab243875 (Lot: 1018144-1), Abcam | WB, IHC, IF, CUT&RUN |
| β-actin | 20536-1-AP, Proteintech | WB |
| c-myc | T55150, Abmart | WB |
| Vimentin | T55134, Abmart | WB |
| Cyclin E1 | 11554-1-AP, Proteintech | WB |
| PCNA | 10205-2-AP, Proteintech | WB |
| Bcl-XL | 10783-1-AP, Proteintech | WB |
| Bax | T40051, Abmart | WB |
| Cleaved-caspase3 | #AF7022, Affinity | WB |
| IGF2BP1 | 22803-1-AP (Lot: 00110774), Proteintech | WB, IF, RIP |
| Nestin | #33475 (Lot: 4), CST | WB, IF |
| m6a | 68055-1-Ig (HP0401), Proteintech | RIP |

**Table S2. RT-qPCR Primer Sequence**

| **Gene** | **Forward (5’-3’)** | **Reverse (5’-3’)** |
| --- | --- | --- |
| Nestin | CTCAGCTTTCAGGACCCCAAG | GCTCAGGACTGGGAGCAAAG |
| Nestin-promoter | ACTTCTGAAAGCCGCCAGAG | AGTTCTGTTCTTGGGGAAGGG |
| SMC1A | CCCAATGGCTCTGGTAAGTCA | ACAATGACACGGGCAAAGGT |
| β-actin | GCACTCTTCCAGCCTTCCTTCC | GCGGATGTCCACGTCACACTTC |
| SMC1A-3’-UTR | GCATCTAGATCATTCACAGCCC | GACTTGGAAGACCTGGGTTCA |

**Table S3. 3C-qPCR Primer Sequence**

| **Gene** | **Sequence (5’-3’)** | **Genecards Enhancer Identifier** |
| --- | --- | --- |
| Nestin promoter | AGTCATCCCTCCAATATCCCCT |  |
| Enhancer#1 | CCCTTTTGTGGTCCTCCAGTT | GH01J156689 |
| Enhancer#2 | ACCACAGGGTCAAGTGGCAG | GH01J156658 |
| Enhancer#3 | TTTCTTCCCAGCTTGTGGTG | GH01J156656 |
| Enhancer#4 | TTCTGGAAATCCTCCAGAGGG | GH01J156648 |
| Enhancer#5 | TCCTGTACAAATGCATAGCACC | GH01J156646 |
| Enhancer#6 | GTCTAGAAGGCAGAAGAGGCT | GH01J156643 |
| Enhancer#7 | AAGACTGAAGGGGACAGAGGAA | GH01J156645 |
| Enhancer#8 | AGTCCATCTTGATCCCTGGG | GH01J156686 |
| Control | TTAGAAGTATGTCCAGCAGGCT |  |

**Table S4. ATAC-qPCR Primer Sequence**

| **Gene** | **Forward (5’-3’)** | **Reverse (5’-3’)** |
| --- | --- | --- |
| Enhancer#1 | AGCAGGGAGAGAGGAATGTGA | GAGTCGTCTCAGGTTTCCTGG |
| Enhancer#2 | GCAATTCTGGAAGCCGTCAG | GTCACCAGCTTGAGTCCCTG |
| Nestin promoter | CGGATGTTTGAACCTCGCAG | TCAGAAAAGGGAGCGACCAG |

**Table S5. Select Assay Sequence**

| **Name** | | **Sequence (5'-3')** |
| --- | --- | --- |
| Probe | Up | tagccagtaccgtagtgcgtgCTCCTGGATCCTCAGGACTTAAG |
|  | Down | 5phos/CCCCAAAGCTCCTTTGTAGCcagaggctgagtcgctgcat |
| Primer | Forward | CCCAATGGCTCTGGTAAGTCA |
|  | Reverse | ACAATGACACGGGCAAAGGT |

**Table S6. Physicochemical characterization**

| PDI | 0.16 ± 0.01 (n = 3) |
| --- | --- |
| Encapsulation efficiency (%) | 91.7 ± 1.5 (n = 3) |
| siRNA loading (wt%) | ~ 1.0 |
| Zeta potential (mV) | −1.8 ± 0.6 (n = 3) |

**Supplementary Table 6 legend**

Polydispersity index (PDI) and zeta potential were measured by dynamic light scattering using a Zetasizer Nano ZS in PBS (pH 7.4). Encapsulation efficiency was determined by a RiboGreen assay. siRNA loading (wt%) was calculated based on the total lipid and siRNA input together with the experimentally determined encapsulation efficiency. Data are presented as mean ± SD from three independent measurements (n = 3).
